# First Report of MPL c.23T>G (p.M8R) Variant in Congenital Amegakaryocytic Thrombocytopenia: A Case Report

**DOI:** 10.1002/jha2.70136

**Published:** 2025-08-28

**Authors:** Atbin Latifi, Sina Yousefian

**Affiliations:** ^1^ School of Medicine Arak University of Medical Sciences Arak Iran; ^2^ Student Research Committee Arak University of Medical Sciences Arak Iran

**Keywords:** case report, congenital amegakaryocytic thrombocytopenia, congenital bone marrow failure syndromes, megakaryocytes, MPL protein, human, thrombocytopenia

## Abstract

Congenital amegakaryocytic thrombocytopenia is a rare inherited bone marrow failure syndrome primarily caused by MPL gene mutations. It presents with severe neonatal thrombocytopenia and typically progresses to pancytopenia. We report the first disease‐associated case of the MPL variant c.23T>G, identified through whole‐exome sequencing in an infant diagnosed with congenital amegakaryocytic thrombocytopenia. Based on ACMG criteria, we propose classification of the MPL c.23T>G (p.M8R) variant as likely pathogenic. This case highlights the importance of early genetic testing in infants with unexplained thrombocytopenia and emphasizes the need to distinguish congenital amegakaryocytic thrombocytopenia from other inherited bone marrow failure syndromes.

**Trial Registration**: The authors have confirmed clinical trial registration is not needed for this submission.

## 1 Introduction

Congenital amegakaryocytic thrombocytopenia (CAMT) is a rare inherited autosomal recessive bone marrow failure (BMF) syndrome. It is characterized by significant thrombocytopenia at birth caused by ineffective megakaryopoiesis, often progressing to BMF and pancytopenia in early childhood [1].

CAMT results from genetic disruptions in the thrombopoietin–MPL signaling axis, essential for megakaryocyte differentiation and hematopoietic stem cell maintenance [2]. Most cases involve mutations in the MPL gene, which encodes the thrombopoietin receptor. Less commonly, mutations in THPO, encoding thrombopoietin itself, are responsible [1]. Defects in this pathway impair megakaryocyte maturation and progressively deplete early hematopoietic progenitors, resulting in BMF and pancytopenia [3].

Clinically, CAMT presents within the first days of life as isolated severe thrombocytopenia, often with platelet counts (PLTs) below 36,000/µL. Newborns may exhibit bleeding symptoms, including petechiae, purpura, rectal, pulmonary, or intracranial hemorrhage [1]. Early bone marrow examinations may appear normal or show reduced megakaryocytes, but megakaryocyte number and maturation progressively decline, with nearly all patients ultimately developing BMF [1].

Here, we report the first clinical association of the MPL c.23T>G (p.M8R) variant with a classical CAMT phenotype, which broadens the known genetic spectrum of this disorder.

## 2 Case Presentation

We report the case of a female neonate who was referred to the pediatric hematology unit shortly after birth for severe thrombocytopenia with a PLT below 20,000/µL. She was born full‐term via vaginal delivery, with no perinatal complications and no family history of hematologic malignancies or disorders. However, parental consanguinity (first cousins) raised suspicion for an underlying genetic etiology. Physical examination was unremarkable, with no bleeding manifestations (e.g., petechiae or purpura) and no dysmorphic features (e.g., microcephaly or limb anomalies) suggestive of a syndromic cause of thrombocytopenia. Initially, neonatal alloimmune thrombocytopenia was suspected. The patient received intravenous immunoglobulin and platelet transfusions, after which PLT rose above 100,000/µL, and she was discharged.

PLT remained above 150,000/µL until 4–5 months of age, when it gradually declined despite the absence of bleeding symptoms (Table 1). WBC and absolute neutrophil count (ANC) remained within normal limits (ANC >1,500/µL), though hemoglobin levels were borderline low with macrocytosis. By 6 months, PLT dropped to 20,000/µL. Bone marrow aspiration and biopsy revealed mild marrow hypoplasia with markedly reduced megakaryocytes and signs of maturation retardation, while other lineages were relatively preserved. A platelet transfusion raised the PLT from 10,000 to 84,000/µL within 30 minutes. The corrected count increment, which accounts for body surface area and platelet dose [4], exceeded 10,000, supporting a platelet production defect rather than peripheral destruction as the cause of thrombocytopenia.

**TABLE 1:** Progressive changes in hematologic parameters from 5 to 9 months of age.

Lab parameter	5 Months	8 Months	9 Months
WBC (×103/µL)	7.2	6.4	4.8
Hb (g/dL)	10.4	9.7	9.8
MCV (fL)	96	100.69	94.43
PLT (/µL)	88,000	33,000	24,000
Neutrophil (%)	35	19	6
Lymphocyte (%)	55	72	88
Monocyte (%)	8	8	4

*Note*: Selected hematologic parameters of the patient, demonstrating a progressive decline in platelet counts starting at 5 months of age following an initially stable postnatal period, with the emergence of macrocytic anemia at 8 months and neutropenia by 9 months. Abbreviations: CAMT, congenital amegakaryocytic thrombocytopenia; Hb, hemoglobin; MCV, mean corpuscular volume; PLT, platelet count; WBC, white blood cell.

By 8 months, the patient developed macrocytic anemia, and by 9 months, she developed leukopenia with ANC below 300/µL (Table 1). A thorough evaluation of BMF syndromes was initiated. Repeat physical examination remained unremarkable, with no growth delay or congenital anomalies (e.g., microcephaly, micrognathia, limb abnormalities, cleft palate, or short stature). Fanconi anemia was excluded by normal chromosomal breakage testing and the absence of renal/skeletal anomalies on imaging. Shwachman–Diamond syndrome was ruled out based on the absence of pancreatic insufficiency or gastrointestinal symptoms. Thrombocytopenia‐absent radius (TAR) syndrome was excluded due to normal radial anatomy, and osteopetrosis was ruled out via normal skull radiographs. Dyskeratosis congenita was not supported by clinical findings.

**FIGURE 1:**
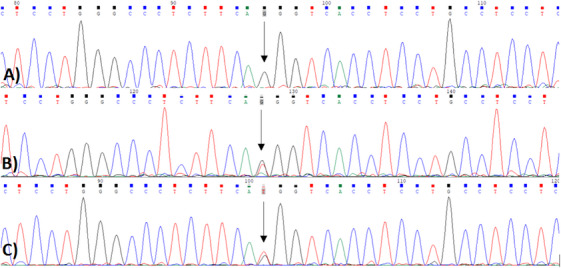
Sanger sequencing chromatograms showing segregation of the MPL c.23T > G (p.Met8Arg) variant in the patient and parents. Sanger sequencing chromatograms of the MPL gene segment containing the c.23T > G (p.Met8Arg) variant in the (A) patient, (B) mother, and (C) father. The patient shows a homozygous G peak at position c.23, while both parents display overlapping T and G peaks consistent with heterozygous carrier status.

Given the consanguineous background, CAMT, a recessive genetic disorder, was suspected. Whole‐exome sequencing identified a previously unreported homozygous missense mutation in the MPL gene: NM_005373.3:c.23T>G (p.Met8Arg). The variant corresponds to a thymine‐to‐guanine substitution at Chr1:43337871 T>G (GRCh38) in exon 1, resulting in the replacement of methionine with arginine at position 8. Sanger sequencing confirmed heterozygous carrier status in both parents, consistent with autosomal recessive inheritance (Figure 1).

The patient was diagnosed with CAMT and underwent hematopoietic stem cell transplantation (HSCT) at 10 months of age from a matched unrelated donor. A myeloablative conditioning regimen was used prior to transplantation, and as of day +34 post‐transplant, the patient demonstrates 100% donor chimerism with normalized hematologic parameters. Adherence to treatment has been complete, and no significant adverse events or issues with tolerability were observed during or after transplantation. The patient remains under close follow‐up.

The patient's guardians expressed satisfaction with the care provided. Informed consent for publication was obtained from the guardians, and identifying details have been omitted per ethical guidelines.

## 3 Discussion

This report presents the first clinical association of the MPL c.23T>G (p.M8R) variant with CAMT. While this variant has been identified once in heterozygous form in the IGSR database (1000 Genomes), it is absent from ClinVar and Genome Aggregation Database (gnomAD) [5], and has not previously been associated with a clinical phenotype.

The clinical course in this patient highlights the diagnostic complexity of CAMT. Two clinical subtypes of CAMT are recognized based on genotype‐phenotype correlation. CAMT I, associated with loss‐of‐function MPL mutations, leads to persistently low PLTs and rapid progression to aplastic anemia. CAMT II, involving mutations with residual MPL activity, typically presents with transient platelet improvement and slower evolution toward pancytopenia [1, 3]. The biphasic clinical course in this case, initial improvement followed by progressive cytopenias, aligns with CAMT type II and underscores the importance of longitudinal monitoring in cases of neonatal thrombocytopenia, especially in consanguineous families.

As an isolated hematologic disorder, CAMT must be distinguished from other inherited BMF syndromes such as Fanconi anemia, dyskeratosis congenita, Shwachman–Diamond syndrome, and TAR syndrome, which also present with early thrombocytopenia but usually include systemic or congenital anomalies [6]. While CAMT typically lacks distinctive physical features, occasional non‐hematologic manifestations involving the brain, eyes, skin, or facial morphology have been reported [1, 3], complicating clinical differentiation. A thorough clinical and genetic evaluation is therefore critical to ensure accurate diagnosis.

The association between the MPL c.23T>G (p.M8R) variant and CAMT in this case is supported by the patient's characteristic CAMT features, the variant's location within the signal peptide domain [7], its autosomal recessive inheritance pattern, and the absence of other plausible pathogenic variants. In silico analysis, using the BLOSUM100 matrix yielded a low substitution score (−4), suggesting the substitution is evolutionarily rare and potentially functionally significant.

The p.Met8Arg substitution lies within the MPL signal peptide [7]. As a type I transmembrane protein, MPL begins with an N‐terminal signal peptide required for co‐translational targeting to the endoplasmic reticulum and membrane insertion [8]. Replacing a hydrophobic methionine with a positively charged arginine may disrupt the signal peptide's hydrophobic core, potentially impairing peptidase cleavage or membrane insertion. This could lead to misfolding, retention in the endoplasmic reticulum, or reduced surface expression of the receptor.

Based on ACMG guidelines [9], the MPL c.23T>G (p.M8R) variant meets the following criteria: PM2 (Extremely rare in population databases [5]), PM3 (homozygous state in the affected child with heterozygous parents), PP4 (highly specific CAMT phenotype), and PP1 (segregation evidence supporting pathogenicity). We propose classifying p.M8R as a likely pathogenic MPL variant and recommend its submission to public variant databases. A limitation of this report is the absence of functional assays to assess the impact of the variant on protein function and downstream signaling.

## 4 Conclusion

This case presents the first clinical association of a homozygous MPL c.23T>G (p.M8R) variant as a likely pathogenic cause of CAMT based on ACMG criteria. Given the phenotypic overlap between CAMT and other inherited BMF syndromes, especially in the early stages, this report emphasizes the importance of early, comprehensive clinical and genetic evaluation in neonates with unexplained thrombocytopenia. Timely diagnosis allows for early referral for HSCT, the only curative treatment for CAMT [1].

To classify the MPL c.23T>G (p.M8R) variant as pathogenic for CAMT, future studies should include functional assays of receptor expression, trafficking, and signaling, in vitro analysis of megakaryocyte differentiation, and expanded segregation and population frequency analyses.

## Author Contributions

**Case conception and design**: A. Latifi, S. Yousefian; **Data collection**: A. Latifi, S. Yousefian; **Analysis and interpretation of results**: A. Latifi, S. Yousefian; **Draft manuscript preparation**: S. Yousefian, A. Latifi; **Supervision**: A. Latifi. All authors reviewed the results and approved the final version of the manuscript.

## Ethics Statement

The study was approved by Arak University of Medical Sciences Ethics Committee (date: 10/30/2024, number: IR.ARAKMU.REC.1403.319). Written informed consent was obtained from the patient's parent for publication of this case report and any accompanying images.

## Consent

Informed consent for publication was obtained from the patient's guardians, and all identifying information was removed to ensure confidentiality according to ethical guidelines.

## Conflicts of Interest

The authors declare no conflicts of interest.

## Supporting information




**Supporting File 1**. jha270136‐sup‐0001‐SuppMat‐WHE.pdf

**Supporting File 2**. jha270136‐sup‐0002‐SupMat‐biopsy.jpg

**Supporting File 3**. jha270136‐sup‐0003‐SupMat‐biopsy‐1.jpg

**Supporting File 4**. jha270136‐sup‐0004‐SupMAt‐Laboratory‐Rresults.jpg

## Data Availability

The data that support the findings of this study are available in the supplementary material of this article. The supplementary file, provided in a zipped format, includes all relevant data. Any personal identifiers or identifying information have been removed from the files to ensure privacy.
