# Correction to “First Report of MPL c.23T>G (p.M8R) Variant in Congenital Amegakaryocytic Thrombocytopenia: A Case Report”

**DOI:** 10.1002/jha2.70148

**Published:** 2025-09-18

A. Latifi and S. Yousefian, “First Report of MPL c.23T>G (p.M8R) Variant in Congenital Amegakaryocytic Thrombocytopenia: A Case Report,” *eJHaem* 6, no. 5 (2025): e70136, https://doi.org/10.1002/jha2.70136.

The authorship statement “Dr. Atbin Latifi shared the first authorship and Dr. Sina Yousefian second authorship.” was incorrect.

This should have read: “Dr. Atbin Latifi and Dr. Sina Yousefian contributed equally and share first authorship.”

We apologize for this error.

