# Supplementary material for: First Report of MPL c.23T>G (p.M8R) Variant in Congenital Amegakaryocytic Thrombocytopenia: A Case Report
Source: EJHaem. 2025 Aug 28;6(5):e70136. doi: 10.1002/jha2.70136 (PMC12393060; doi:10.1002/jha2.70136)
Supplement: Supplementary file 1 — Supporting File 1. jha270136‐sup‐0001‐SuppMat‐WHE.pdf [file JHA2-6-e70136-s002.pdf]

Report status: Primary

SAMPLE INFORMATION

Patient Name: [redacted]	GeneFan Accession No: gfan6128
Date of Birth: 1-year-old/female	Date Specimen Obtained:
Specimen Type: Blood	Date Specimen Received:
Submitters ID No:	Date of Report: 24 September 2024

CLINICAL SYMPTOMS OF THE PATIENT

An affected 1 year old female with related parents. She has Congenital Thrombocytopenia. The baby born with ecchymosis. Systemic workup revealed no coagulation disorders. Bone marrow examination revealed hypoplasia of megakaryocytes. Other bone marrow elements and general cellularity was normal.

Result

Panels such as Blood Disorders were considered for this subject.

UNCLEAR RESULT
Variants of uncertain significance (VUS) identified

RECOMMENDATION

- Genetic counselling is recommended.

MAIN RESULTs: Actionable variant (Pathogenic, Likely pathogenic, VUS)

Gene	Position/Variant (hg38)	Zygosity	Inheritance	Disease	Allele Frequency (gnomAD)	Classification
MPL	chr1:43337871:T>G: NM_005373:exon1:c.23T>G:p.M8R	HOM	AR	Thrombocytopenia،congenital amegakaryocytic (604498)	0.000007	**Uncertain Significance**

The *MPL* gene encodes the receptor for thrombopoietin (THPO; 600044), a hematopoietic growth factor that regulates the production of multipotent hematopoietic progenitor cells and platelets.

Congenital amegakaryocytic thrombocytopenia-1 (CAMT1) [604498] is an autosomal recessive disorder characterized by ***onset of thrombocytopenia and megakaryocytopenia in infancy*** or early childhood. The disorder is progressive and evolves to pancytopenia and bone marrow failure. Serum thrombopoietin is elevated. There is a favorable response to bone marrow transplantatio

The NM_005373.3(MPL):c.23T>G(p.Met8Arg) variant causes a missense change involving the alteration of a non-conserved nucleotide. The variant allele was found at a frequency of 0.00000657 in 152,200 control chromosomes in the GnomAD database, with no homozygous occurrence. Variant has been reported in Lovd as Likely pathogenic (no stars). Another variant affecting the same amino acid position, but resulting in a different missense (i.e. M8V) has been classified as Uncertain significance. Therefore, based on ACMG classification, this variant is Uncertain Significance.

Comments:
The c.23T>G variant in *MPL* gene located in a unique region with no duplication or pseudogene sequences, however repetitive structures exist in its flanking regions.

**Incidental Finding:** ACMG SF v3.2 (PubMed 37347242)						
**Gene**	**Position/Variant (hg38)**	**Zygosity**	**Inheritance**	**Disease**	**Allele Frequency (gnomAD)**	**Classification**
-	-	-	-	-	-	-
**Comments:**<br>No variant was detected.						

**CNV (exon-loss) Variation:**				
**Gene**	**Position/Variant (hg38)**	**Disease**	**Type**	**Clinical Significance**
-	-	-	-	-
**Comments:**<br>-				

Supplementary						
**Gene**	**Position/Variant (hg38)**	**Zygosity**	**Inheritance**	**Disease**	**Allele Frequency (gnomAD)**	**Classification**
-	-	-	-	-	-	-

Comments:
No variant was detected.

References: The following references are used in variant analysis and classification when applicable for observed genetic alterations.

1. The 1000 Genomes Project Consortium. An integrated map of genetic variation from 1092 human genomes. Nature. 2012;491:56-65.
2. Standards and guidelines for the interpretation of sequence variants. Genet Med. 2015 May;17(5):405-23.
3. Ambry Genetics Variant Classification Scheme. http://www.ambrygen.com/variant-classification.
4. Database of Single Nucleotide Polymorphisms (dbSNP) [Internet]. Bethesda (MD): National Center for Biotechnology Information, NationalLibrary of Medicine (dbSNP Build ID:135) Available from: www.ncbi.nlm.nih.gov/SNP. Accessed Jan 2012).
5. ESEfinder [Internet]. Smith PJ, et al. (2006) Hum Mol Genet. 15(16):2490-2508 and Cartegni L, et al. Nucleic Acid Research. 2003;31(13):3568-3571. http://rulai.cshl.edu/cgi-bin/tools/ESE3/esefinder.cgi?process=home.
6. Exome Variant Server, NHLBI Exome Sequencing Project (ESP) [Internet], Seattle WA. Available from: evs.gs.washington.edu/EVS.
7. Grantham R. Amino acid difference formula to help explain protein evolution. Science. 1974;185(4151):862-864.
8. HGMD® [Internet]: Stenson PD et al. Genome Med. 2009;1(1):13. www.hgmd.cf.ac.uk.
9. Landrum MJ et al. ClinVar: public archive of relationships among sequence variation and human phenotype. Nucleic Acids Res. 2014 Jan1;42(1):D980-5. doi: 10.1093/nar/gkt1113. PubMed PMID: 24234437.
10. Online Mendelian Inheritance in Man, OMIM®. McKusick-Nathans Institute of Genetic Medicine, Johns Hopkins University (Baltimore, MD), Copyright® 1966-2012. World Wide Web URL: http://omim.org.
11. PolyPhen [Internet]: Adzhubei IA, et al. Nat Methods. 2010;7(4):248-249. genetics.bwh.harvard.edu/pph2.
12. SIFT [Internet]: Ng PC & Henikoff S. Hum Genet. 2006;7:61-80. http://sift.jcvi.org.
13. Exome Aggregation Consortium (ExAC) [Internet], Cambridge, MA. Available from: http://exac.broadinstitute.org.
14. Genome Aggregation Database (gnomAD) [Internet], Cambridge, MA. Available from: http://gnomad.broadinstitute.org.
15. King, S., Germeshausen, M., Strauss, G., Welte, K., Ballmaier, M. Congenital amegakaryocytic thrombocytopenia: a retrospective clinical analysis of 20 patients. Brit. J. Haemat. 131: 636-644, 2005.

REPORT ELECTRONICALLY SIGNED BY:

**Dr. Hamzeh Rahimi**<br>PhD of Medical Biotechnology	**Dr. Bahadory, M.D**<br>Consultant Clinical Geneticist

Supportive Information and Glossary

Incidental Finding

A subset of pharmacogenetic variants, and sequence variants in a subset of non-clinical feature related genes (based on ACMG incidental finding guideline 20xx), were also identified by this assay as incidental findings and are reported below. Only currently known pathogenic mutations are reported here, variants of uncertain significance are not reported. Sequencing technology is continually evolving, and the interpretation of genetic findings may change over time. If you believe your patient has a genetic condition, evaluation by a genetic specialist to determine the need for additional genetic testing may be warranted.

Structural variation (SV)

Structural variation (SV) is generally defined as a region of DNA approximately 1 kb and larger in size and can include inversions and balanced translocations or genomic imbalances (insertions and deletions), commonly referred to as copy number variants (CNVs). These CNVs often overlap with segmental duplications, regions of DNA >1 kb present more than once in the genome, copies of which are >90% identical. If present at >1% in a population a CNV may be referred to as copy number polymorphism (CNP).

Supportive Information and Glossary

Result Reports: In result reports, alterations in the following classifications are always reported, and are based on the following definitions and clinical recommendations:

Pathogenic Mutation: Alterations with sufficient evidence to classify as pathogenic (capable of causing disease). Targeted testing of at-risk relatives and appropriate changes in medical management for pathogenic mutation carriers are recommended.

Variant, Likely Pathogenic (VLP): Alterations with strong evidence in favor of pathogenicity. Targeted testing of at-risk relatives and appropriate changes in medical management for VLP carriers typically are recommended. Previously described likely pathogenic variants, including intronic VLPs at any position, are

always reported when detected.

Variant, Unknown Significance (VUS): Alterations with limited and/or conflicting evidence regarding pathogenicity. Familial testing via the Family Studies Program is recommended. Medical management to be based on personal/family clinical histories, not VUS carrier status. Note, intronic VUSs are always reported out to 5 basepairs from the splice junction when detected.

Alterations of unlikely clinical significance (those with strong/very strong evidence to argue against pathogenicity) are not routinely included on results reports. These include findings classified as “likely benign” and “benign” alterations.

Methodology

Genomic deoxyribonucleic acid (gDNA) is isolated from the patient’s specimen using a filter-based methodology and quantified. A total amount of 1.0μg genomic DNA per sample was used as input material for the DNA sample preparation. Sequencing libraries were generated using Agilent SureSelect Human All ExonV7 kit (Agilent Technologies, CA, USA) following manufacturer’s recommendations and x index codes were added to attribute sequences to sample. Briefly, fragmentation was carried out by hydrodynamic shearing system (Covaris, Massachusetts, USA) to generate 180-280bp fragments. Remaining overhangs were converted into blunt ends via exonuclease/polymerase activities and enzymes were removed. After adenylation of 3’ ends of DNA fragments, adapter oligonucleotides were ligated. DNA fragments with ligated adapter molecules on both ends were selectively enriched in a PCR reaction. Captured libraries were enriched in a PCR reaction to add index tags to prepare for hybridization. Products were purified using AMPure XP system (Beckman Coulter, Beverly, USA) and quantified using the Agilent high sensitivity DNA assay on the Agilent Bioanalyzer 2100 system. The qualified libraries are fed into NovaSeq 6000 Illumina sequencers. Then data quality control, analysis and interpretation were run on the G9 generation of HP server using a unix based operating system.

COMMENTS

This assay was developed and its performance determined for the sole purpose of identifying small sequence variants in the coding regions tested. This test may not detect large chromosomal aberrations, such as larger deletions and duplications or rearrangements. Normal findings do not rule out the diagnosis of any disorder since some genetic abnormalities may be undetectable with this assay. The SureSelect V7-postcap kit does not target all coding exons of all known RefSeq genes; the genomic coordinates of the regions not covered are available on request.

Disclaimer:

- This test should be interpreted in context with other clinical findings. Any questions, suggestions, or concerns regarding interpretation of results should be forwarded to a genetic counselor, medical geneticist, or physician skilled in interpretation of the relevant medical literature.
- The whole exome sequencing test analyzes the following types of mutations: nucleotide substitutions, small deletions (up to 25 bp), small insertions (up to 10 bp) and small indels. Other than alterations noted in the methodology section above, these assays are not intended to analyze the following types of mutations: gross rearrangements, deep intronic variations, Alu element insertions, and other unknown abnormalities. The pattern of mutation types varies with the gene tested and this test detects a high but variable percentage of known and unknown mutants of the classes stated. A negative result from the analysis cannot rule out the possibility that the tested individual carries a rare unexamined mutation or mutation in the undetectable group.
- The WES test is designed and validated to be capable of detecting >85% of described mutations in the genes represented on the tests (analytical sensitivity). The clinical sensitivity of the WES test may vary widely according to the specific clinical and family history.
- Although molecular tests are highly accurate, rare diagnostic errors may occur. Possible diagnostic errors include sample mix-up, erroneous paternity identification, technical errors, clerical errors, and genotyping errors. Genotyping errors can result from trace contamination of PCR reactions, from maternal cell contamination in fetal samples, from rare genetic variants that interfere with analysis, low-level mosaicism, presence of pre-malignant or malignant cells in the sample, presence of pseudogenes, technical difficulties in regions with high GC content or homopolymer tracts, or from other sources. Rare variants present in the human genome reference sequence (GRCh38.p13/hg38) or rare misalignment due to presence of pseudogenes can lead to misinterpretation of patient sequence data.
- The risk of technical errors of DNA analysis is estimated to be 0.5%. The risk of error from DNA recombination in diagnosis by polymorphism is also approximately 0.3%.
- Laboratories are required to provide the company with all clinical information about the proband and other patients in the family. Otherwise, the Company is not responsible for possible consequences such as failure to achieve appropriate disease-related results.
- All sections of this report are interpreted together and cannot be interpreted separately.

GenomeFan *Pishro Teb* - Unit 5, No. 23, 2d Keihan Alley, Keihan St., Ayatollah Kashani Blvd., Sadeghieh, Tehran, Iran
[illegible]
