# Supplementary figures and images for: First Report of MPL c.23T>G (p.M8R) Variant in Congenital Amegakaryocytic Thrombocytopenia: A Case Report
Source: EJHaem. 2025 Aug 28;6(5):e70136. doi: 10.1002/jha2.70136 (PMC12393060; doi:10.1002/jha2.70136)

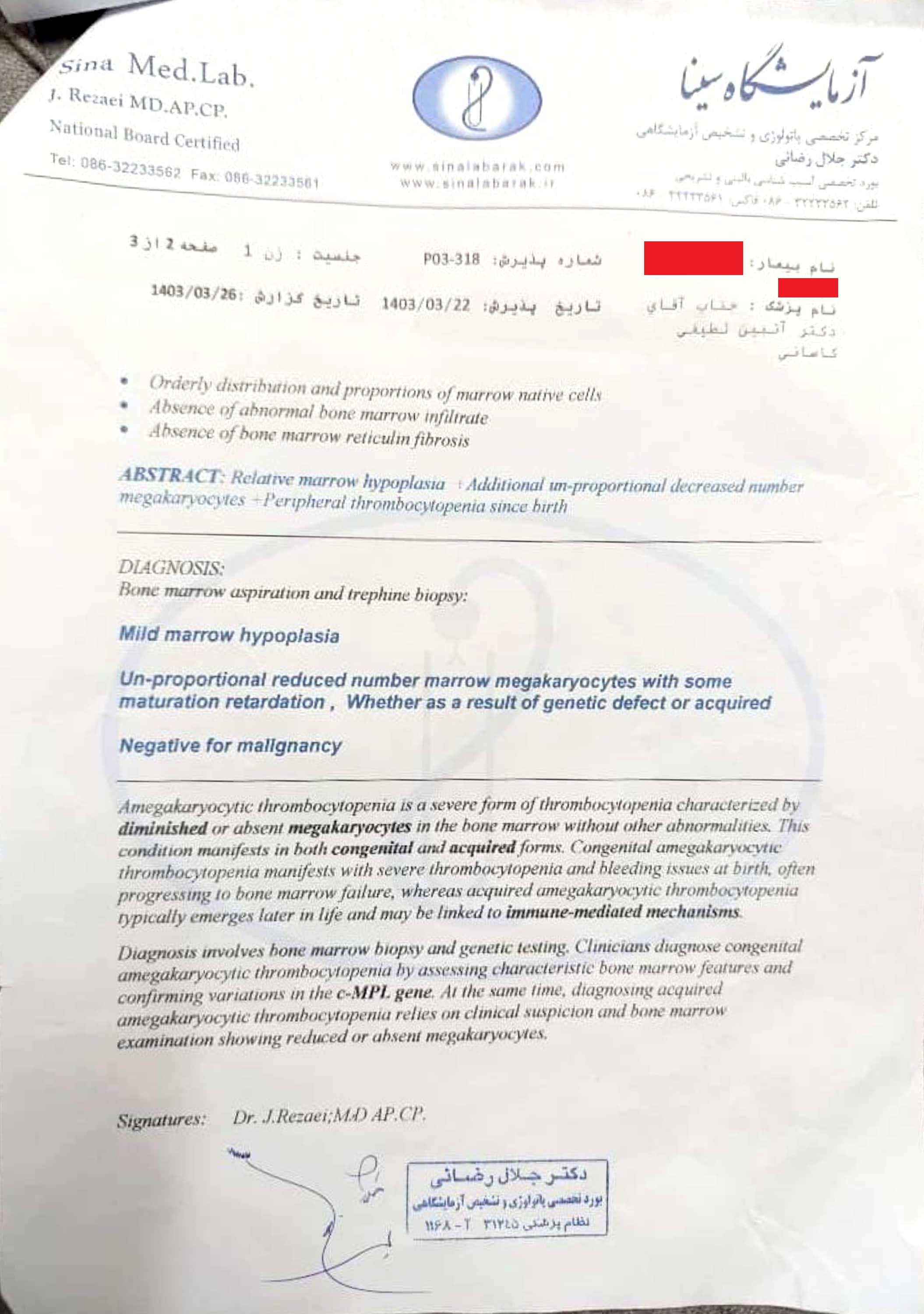

Supplement: Supplementary file 2 — Supporting File 2. jha270136‐sup‐0002‐SupMat‐biopsy.jpg [file JHA2-6-e70136-s003.jpg]

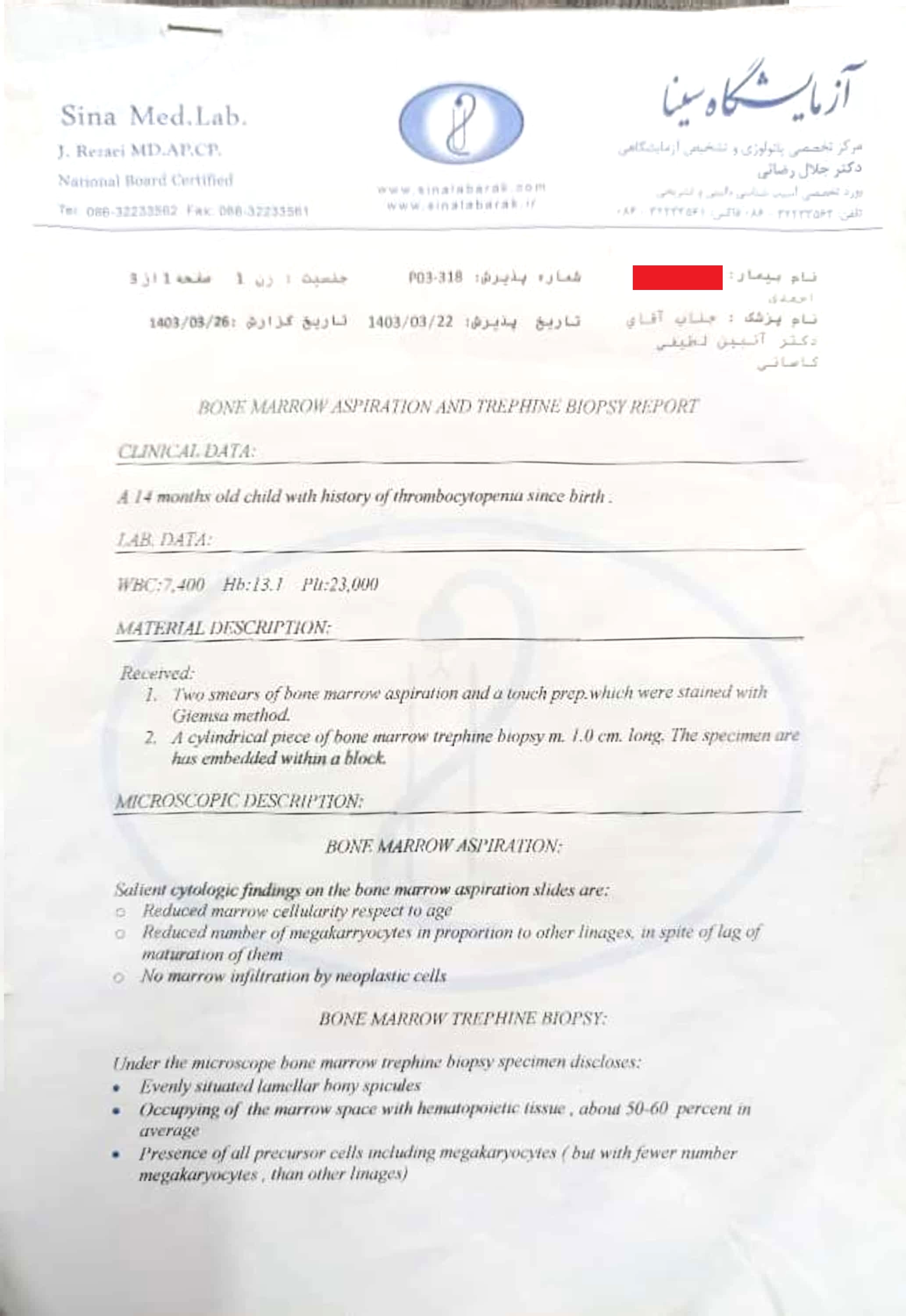

Supplement: Supplementary file 3 — Supporting File 3. jha270136‐sup‐0003‐SupMat‐biopsy‐1.jpg [file JHA2-6-e70136-s004.jpg]

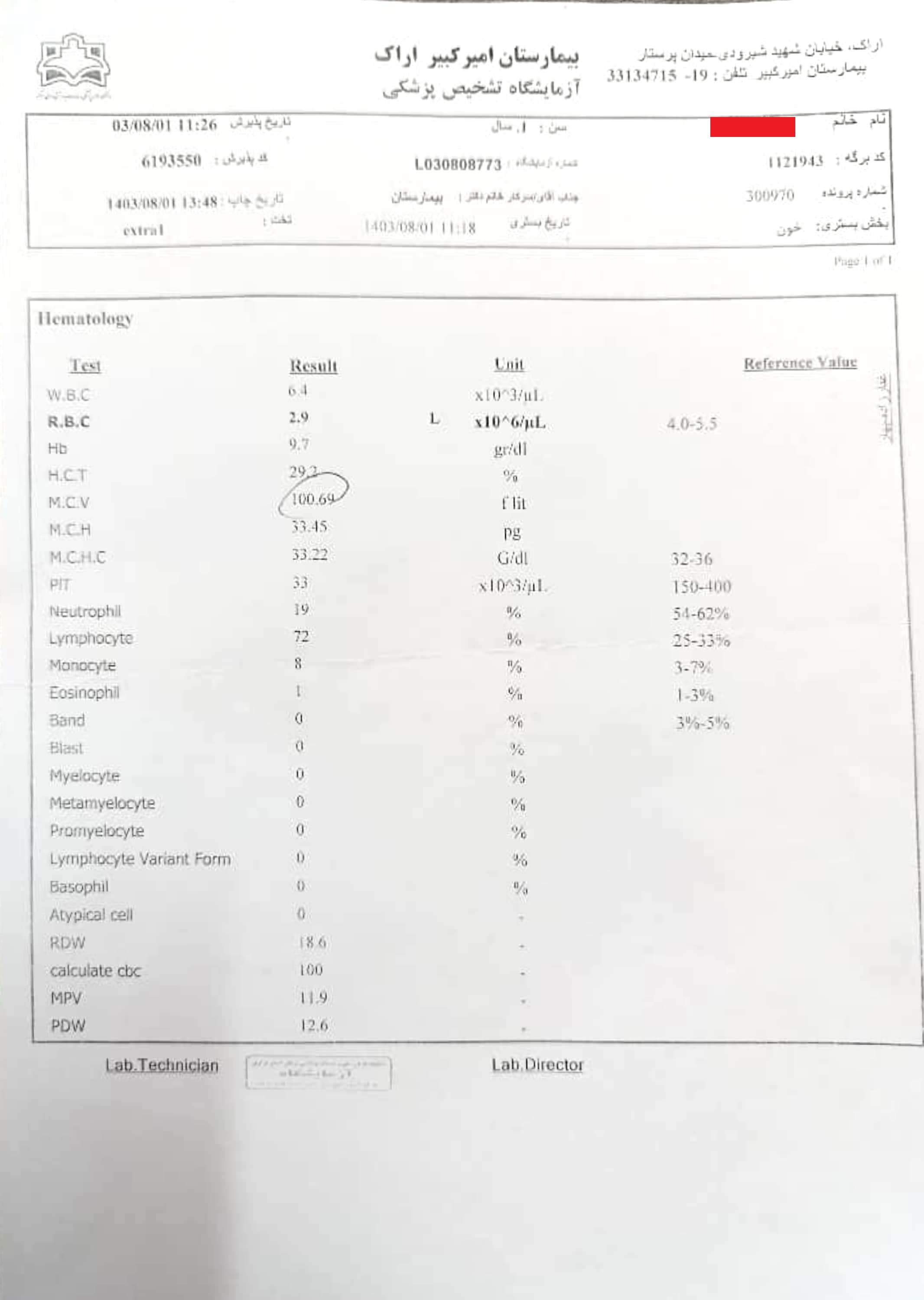

Supplement: Supplementary file 4 — Supporting File 4. jha270136‐sup‐0004‐SupMAt‐Laboratory‐Rresults.jpg [file JHA2-6-e70136-s001.jpg]
